# Comparison the effect of Asvard capsule and omeprazole in treatment and recurrence of non-cardiac chest pain related to gastroesophageal reflux disease in adults: A controlled double blind clinical trial

**DOI:** 10.22038/ajp.2025.25580

**Published:** 2025

**Authors:** Saeed Sepehrikia, Nasser Behnampour, Ayesheh Enayati, Alireza Norouzi, Fatemeh Kolangi

**Affiliations:** 1 *Golestan Research Center of Gastroenterology and Hepatology, Department of Persian Medicine, School of* *Persian Medicine, Golestan University of Medical Sciences, Gorgan, Iran*; 2 *Biostatistics Department,Faculty of Health,Golestan University of Medical Sciences,Gorgan, Iran*; 3 *Ischemic Disorders Research Center, Golestan University of Medical Sciences, Gorgan, Iran*; 4 *Golestan Research Center of Gastroenterology and Hepatology, Golestan University of Medical Sciences, Gorgan, Iran*; 5 *Counseling and Reproductive Health Research Centre,Department of Persian Medicine, School of Persian Medicine, Golestan University of Medical Sciences, Gorgan, Iran*

**Keywords:** Herbal medicine, Gastroesophageal reflux, Non-cardiac chest pain, Complementary medicine Persian medicine

## Abstract

**Objective:**

Considering the burden of non-cardiac chest pain (NCCP) on emergency departments and the difficulties related to the treatment of the disorder, the present study aims to investigate the effect of Asvard capsule (containing *Myrtus communis* and *Rosa damascena*) compared to routine treatment (omeprazole) in a double-blind randomized controlled clinical trial.

**Materials and Methods:**

This study was carried out for 8 weeks including 6 weeks of therapeutic intervention and 2 weeks of follow-up. The participants were assigned to a control group that received omeprazole capsule 20 mg/day and three intervention groups A) Asvard capsule 1200 mg/day )2 cap 300 mg/BID), B) Asvard capsule 1600 mg/day (2 cap 400 mg/BID), and C) Asvard capsule 2000 mg/day (2 cap 500 mg/BID). The therapeutic efficiency for NCCP was assessed in 4 phases (days 0, 21, 42, and 56) using a standardized questionnaire of frequency scale for the symptoms of gastroesophageal reflux disease (FSSG).

**Results:**

The average value of NCCP was reduced after the intervention compared to the study's baseline in all groups (Pv <0.001). However, two weeks after treatment (follow-up), participants recorded higher scores of NCCP in the omeprazole 20 mg group, the average of NCCP for the intervention groups remained constant or increased slightly.

**Conclusion:**

Our findings present that although omeprazole 20 mg decreased chest pain score, it has less lasting effect compared to the herbal medicine used in this study. It seems that Asvard can be effective for the treatment of NCCP related to gastroesophageal reflux disease (GERD), however, a long-term study

## Introduction

Non-cardiac chest pain (NCCP) is a prevalent complication (Ramesh et al. 2021). For example, in different regions of the world, the incidence of NCCP is estimated at 10 to 30% (Stepinska et al. 2020) and in America, 80 million people are affected by NCCP every year (Nawar et al. 2007). This complication is more commonly known as a problem related to gastroesophageal reflux disease (GERD) (Park et al. 2015). This complication causes an additional burden on emergency departments, reducing the quality of life of patients and causing disability. It is difficult and expensive to diagnose and differentiate NCCP from chest pain associated with heart problems (McDevitt-Petrovic et al. 2017). Clinical findings such as ECG, Troponin 1 test, and blood pressure are known as methods of distinguishing NCCP from heart problems (Mateen et al. 2024).

The incidence of NCCP varies according to geographic location, demographic, and clinical factors. Similar to GERRD, the role of various factors such as lifestyle and psychological disorders has been reported as the risk factors of NCCP. In addition, NCCP rarely has a different risk factor profile in people without reflux (Gonzalez-Ibarra et al. 2024; Saitta and Hebbard 2022; Wise et al. 2005).

Etiologically, chest pain with non-cardiac origin can be caused by respiratory causes such as pleural disease, pneumothorax, pulmonary embolism, pneumonia, neurological causes such as neuromuscular disorders, and digestive ones such as GERD, hiatal hernia, esophageal motility disorders, esophageal hypersensitivity (Achem 2008; Cannata’ et al. 2019). However, GERD is the main cause of NCCP; in 66% of cases, NCCP and GERD are observed as comorbidities, and only 30% of NCCP cases are not related to GERD (Dickman et al. 2007). NCCP associated with GERD can be a serious alarm about advanced esophageal problems; about one-third of these cases have abnormal upper gastroenterology (UGI) endoscopy (Abdi et al. 2018). Both NCCP and GERD are among the primary manifestations in Barrett's esophagus and esophageal adenocarcinoma patients (Oldfield Iv et al. 2021). Most often, treatment of GERD also leads to improvement of NCCP (Oldfield Iv et al. 2021; Sanchez and Mehta 2021).

For example, the treatment of GERD using medications such as Proton pump inhibitors (PPIs) was effective in controlling NCCP. According to the results of a systematic review, the use of PPIs -omeprazole 20 mg/day and ranitidine 150 mg/day- has improved about 80 to 100% of NCCP cases, respectively (Hershcovici et al. 2012). However, the use of medications has two major weaknesses: side effects and the possibility of recurrence. Therefore, recently, herbal medicine to control digestive problems has received much attention. Two herbal compounds frequently used to improve digestive disorders are the extract of *Myrtus communis* and *Rosa damascena*. These two herbs have been used separately in several studies with positive effects on the treatment of digestive problems such as GERD with the least side effects (Adel Mehraban et al. 2021; Zohalinezhad et al. 2016), however, studies aimed at investigating the effect of herbal medicines on NCCP are very limited. Therefore, the present study was conducted to assess the efficiency of Asvard capsules containing the extracts of *M. communis* and *R. damascena* on the NCCP complication related to GERD compared to routine treatment (omeprazole) in a double-blind clinical trial.

## Materials and Methods

This study was a double-blind randomized controlled clinical trial conducted in four groups including one control and three intervention groups. The participants in the study were patients with confirmed symptomatic reflux who were referred to the medical centers of Golestan University of Medical Sciences. The final diagnosis of GERD and its related symptoms was ultimately made by a gastroenterologist using diagnostic criteria and clinical tests. After taking a history, completing the ROME IV questionnaire, performing an endoscopy, and checking the results of the *H. pylori* and clinical tests, patients who had functional and non-organic reflux and met the inclusion criteria were enrolled in the study. 

### Inclusion and exclusion criteria

The included participants were literate patients aged 18-60 years, diagnosed with GERD based on ROME IV criteria and the results of endoscopy and clinical tests, without specific pathological findings such as *H. pylori* infection (Feldman et al. 1998). They did not have a history of smoking, alcohol consumption, diabetes, thyroid disorders, or heart, kidney or liver failure. Also, the participants in the study had not taken antibiotics, NSAIDs, or aspirin within 1 month before the start of the study, the onset of their symptoms was 6 months, and they had GERD-related problems or symptoms at least 2 days a week in the last three months.

Exclusion criteria were 1) taking any medication with a direct effect on the digestive system during the study period, 2) consuming less than 80% of the medication doses by the patient, 3) having any allergies, persistent disorder, or symptom related to the medicine, and 5) not completing the treatment or follow-up steps of the study.

### Blinding and patients' allocation

Participants in the study were allocated to four groups using the block randomization method. The size of the blocks was equal to 8 and each block had four letters, B, C, D, or A and each letter had two repetitions; these blocks could be as follows: CBDCABDA, DCBACADB, ABBDCDAC, BACDDABC, etc. Based on randomization, we ultimately had 18 blocks with 8 letters, totaling 144 patients randomly assigned into the groups. 

For allocation concealment, patients based on their selected code, received a medicine package, without any direction. Patients and medicine givers did not know the content of the treatment packages. Asvard and omeprazole capsules were identical in terms of physical shape, packaging, and label type. The Persian language prescription for the medication was placed in the packages. Besides, the telephone number of a physician who was aware of the study process was provided at the end of the prescription, and patients could contact him if necessary to report side effects or ask questions. However, the physician had no role in medicine distribution, data analysis, and patient allocation. 

### Herbal medicine preparing

To prepare Asvard capsules, 1 kg of each plant (*M. communis* and *R. damascena*) was purchased from the market. After washing, each plant was soaked in 10 l of ethanol 70% for 24 hr at room temperature and then stirred for 8 hr at 45 ^o^C in a cooking pot with a mixer. Then, the extract of plants was separated from ethanol in a pot under a negative pressure of 7 bars, and a temperature of 65 ºC. Finally, 1 kg of liquid extract with a concentration of 10 kg/L was collected, then the liquid extract was powdered with a spray dryer at a temperature of 190 ºC and 100 g of concentrated and pure powder was obtained. After confirming the physicochemical and microbial tests, the powder of both plants with a ratio of 3:1 (Myrtle/ Rose) was encapsulated. We provided three doses for the Asvard capsules used in this research; 300 mg (225 mg of Myrtle and 75 mg of Rose), 400 mg (300 mg of Myrtle and 100 mg of Rose), and 500 mg (375 mg of Myrtle and 125 mg of Rose). Herbal medicine preparations were carried out in a plant company. The prescription monitoring program (PMP) codes of the plants were received from the Faculty of Pharmacy in Tehran, which were PMP-1526 and PMP-4629 *M. communis* and *R. damascena*, respectively. The doses of the herbal medicine were prepared based on reference of Persian Medicine - Makhzan Al- Advia (Aghili) (Group 2020). 

### Intervention and patient evaluation

The study lasted 8 weeks including 6 weeks of intervention and the 2 weeks of follow-up (Table 1). During the study period, treatment results were recorded using a questionnaire. The main outcome measured in the study was NCCP. The data was obtained using a standardized questionnaire of frequency scale for the symptoms of gastroesophageal reflux disease (FSSG), in which the answers to the questions are on a 4-point scale from 0 to 4; the higher the score, the more frequent and severe complications (Kusano et al. 2004; Zohalinezhad et al. 2016). The questionnaire was filled on days 0, 21, 42, and 56 (baseline, the end of the third week, the end of the intervention, and 2 weeks after the end of the intervention, respectively). In addition to FSSG questionnaire, demographic survey questions were asked using another questionnaire on the baseline of the study. Also, side effects of treatment methods were fully recorded by the physician who was in connect with the patients.

### Ethical considerations

The present study was ethically reviewed and approved by the ethics committee of Golestan University of Medical Sciences (IR.GOUMS.REC.1401.555), and registered on the Iranian clinical trial website with the code IRCT20230226057538N1. All the patients were asked to fill informed consent form.

### Sample size and statistical analysis

The sample size with a confidence level of 95% and a test power of 80% (α=0.05 and β2=0) and considering the possibility of 15% dropout, was determined to be 36 patients in each group.

Data analysis was carried out using Related-Samples Friedman's Two-Way and analysis of Variance test, ranks and significance values ​​have been adjusted by the Bonferroni correction for multiple tests and Generalized estimating equations (GEE), in R environment version 4.0.2. The significance level of all statistical tests was 0.05.

**Table 1:** The four groups included in this trial.

**Group**	**Medication**	**Type of capsule**	**Daily dose**	**Prescription**
Control	Omeprazole	Omeprazole 20 mg capsule	20 mg	Every morning before fast-breaking
Intervention A	Asvard 1200 mg per day	Asvard 300 mg capsule	900 mg of *M. communis* + 300 mg of *R. damascena*	After breakfast and dinner (twice a day and 2 capsules each time)
Intervention B	Asvard 1600 mg per day	Asvard 400 mg capsule	1200 mg of *M. communis* + 400 mg of *R. damascena*	After breakfast and dinner (twice a day and 2 capsules each time)
Intervention C	Asvard 2000 mg per day	Asvard 500 mg capsule	1500 mg of *M. communis* + 500 mg of *R. damascena*	After breakfast and dinner (twice a day and 2 capsules each time)

## Results

**Figure 1:**
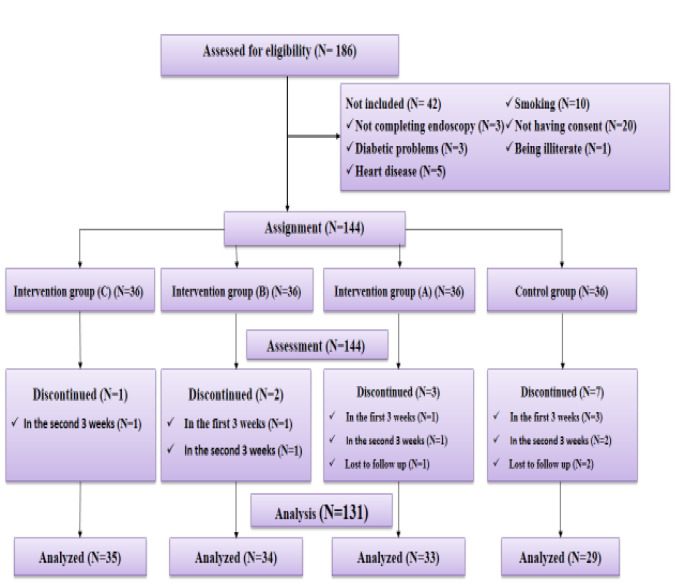
CONSORT diagram of study population

One hundred thirty-one patients, 30 women and 101 men, completed the study (Figure 1). Concerning the background variables, patients who left the study were not significantly different from those who remained in the groups. Also, all the groups were matched in age and gender and had no statistically significant difference (p >0.05).

Our findings indicated that the NCCP score was reduced in all control and intervention groups, however, the effect of the four treatment protocols was not the same during the study period. The average of the NCCP score was reduced in all groups in the treatment phase of the study. The highest measures of the NCCP, determining worse disorder conditions, in all phases of the study were recorded for the control group. 

**Figure 2:**
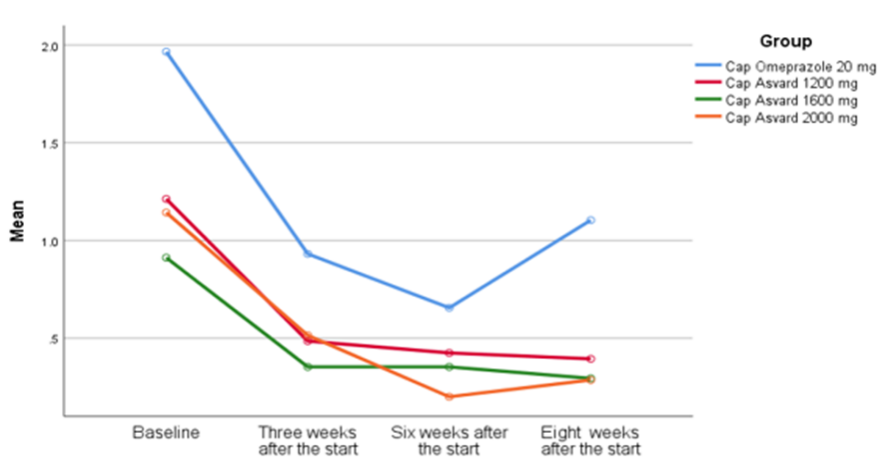
Comparison of the average score of NCCP in the four investigated groups during the study period

The results presented in Table 2 demonstrate that the average score of NCCP had remarkably reduced between baseline and the end of the third week of the intervention, however, there was no significant change between the third and sixth weeks in all the groups. Two weeks after ending the intervention, follow-up phase, the chest pain score significantly increased in the omeprazole group, however, it remained constant or had a slight change in the intervention groups (Figure 2). 

Comparing the average score of NCCP in all groups by GEE test showed a statistically significant difference between the omeprazole group and the three intervention groups (p <0.036). In other terms, the average score of NCCP in intervention groups was significantly lower than that of the omeprazole group (Table 3).

Side effects were observed in only a limited number of patients. The most frequent side effect was constipation presented by participants in all groups. In addition, six participants (three in group B and three in intervention group C) had nausea. Other side effects such as heaviness in stomach, xerostomia, headache, and poor appetite were reported in limited cases (Table 4).

**Table 2:** Mean ± Standard deviation for chest pain between omeprazole and other interventions

**Group**	**Time**				p-Value*
	**Baseline**	**Three weeks after the start**	**Six weeks after the start**	**Eight weeks after the start**	
Cap Omeprazole 20 mg	1.97± 1.476	0.93± 1.334	0.66± 1.289	1.10± 1.423	<0.0001
Cap Asvard 1200 mg	1.21±1.364	0.48±0.972	0.42±0.867	0.39±0.704	<0.0001
Cap Asvard 1600 mg	0.91±1.190	0.35±0.884	0.35±1.012	0.29±0.836	<0.0001
Cap Asvard 2000 mg	1.14±1.192	0.51±0.781	0.20±0.584	0.29±0.622	<0.0001

*Related-Samples Friedman's Two-Way Analysis of Variance test by Ranks

**Table 3:** Pairwise comparisons within group

**Group**	**Sample 1**	**Sample 2**	p-Value*
Cap Omeprazole 20 mg	Baseline	Three weeks after the start	0.004
	Baseline	Six weeks after the start	0.001
	Baseline	Eight weeks after the start	0.025
	Three weeks after the start	Six weeks after the start	0.542
	Three weeks after the start	Eight weeks after the start	0.524
	Six weeks after the start	Eight weeks after the start	0.222
Cap Asvard 1200 mg	Baseline	Three weeks after the start	0.019
	Baseline	Six weeks after the start	0.012
	Baseline	Eight weeks after the start	0.017
	Three weeks after the start	Six weeks after the start	0.849
	Three weeks after the start	Eight weeks after the start	0.962
	Six weeks after the start	Eight weeks after the start	0.886
Cap Asvard 1600 mg	Baseline	Three weeks after the start	0.039
	Baseline	Six weeks after the start	0.037
	Baseline	Eight weeks after the start	0.024
	Three weeks after the start	Six weeks after the start	0.999
	Three weeks after the start	Eight weeks after the start	0.854
	Six weeks after the start	Eight weeks after the start	0.851
Cap Asvard 2000 mg	Baseline	Three weeks after the start	0.042
	Baseline	Six weeks after the start	0.001
	Baseline	Eight weeks after the start	0.004
	Three weeks after the start	Six weeks after the start	0.179
	Three weeks after the start	Eight weeks after the start	0.379
	Six weeks after the start	Eight weeks after the start	0.643

*Post Hoc test after Friedman's Two-Way Analysis of Variance

**Table 4:** The number of participants who reported side effect in each group.

**Groups**	**The number of participants reported side effects**						
	Nausea (N)	Constipation (N)	Bellyache (N)	Heaviness in stomach (N)	Xerostomia (N)	Headache (N)	Poor appetite (N)
Control	-	2	-	-	-	-	-
Intervention A (Asvard 1200 mg/d)	-	2	1	2	1	-	-
Intervention B (Asvard 1600 mg/d)	3	2	-	2	-	2	-
Intervention C (Asvard 2000 mg/d)	3	1	-	-	-	-	1

## Discussion

Non-cardiac chest pain (NCCP) is a common complication in patients with reflux or GERD. NCCP has been observed in 60% of GERD patients (Saitta and Hebbard 2022). Approximately one-fourth of people are affected by NCCP during their lifetime (Fass and Achem 2011). This complication is known as a very low-mortality disorder (less than 3%), however, the burden of it is high, mainly due to the inability to work and use of health care services, especially emergency department (Foldes-Busque et al. 2024; Mol et al. 2018). Several pathophysiological mechanisms including gastroesophageal reflux disease (GERD), esophageal motility disorders, psychiatric disorders, abnormal brain processing of abdominal stimulation, and impaired autonomic activity have been introduced as the causes of NCCP. Early diagnosis and treatment of chest pain is very important, especially in severe cases. Severe NCCP is sometimes confused with heart problems. Even in some types of cancer, severe NCCP is known as an alarm of the beginning of a malignancy or metastasis (Bima et al. 2023). In addition, chest pain is a substantial symptom when it is accompanied by reflux, in such cases, chest pain can be a symptom of gastrointestinal cancers and carcinoma polyps (Shakhatreh et al. 2015; Xie et al. 2021). NCCP related to GERD could be treated by lifestyle modification and pharmacological interventions. Elevating 

the head of the bed, reducing fat intake, quitting smoking, and avoiding fatty and greasy foods could alleviate reflux symptoms (Kim and Rhee 2012). 

PPIs are the most effective and widely used medical intervention for the treatment of GERD and its symptoms such as NCCP. Earlier studies presented that PPIs have better medical achievement than Histamine H2-receptor antagonists (H2RAs) for caring for esophageal mucosa and relieving NCCP in patients with GERD. For example, a meta-analysis showed that the therapeutic efficacy of PPIs was significantly better than H2RAs (83.6% vs. 51.9%) after 12 weeks of intervention (Chiba et al. 1997). The same effect on heartburn severity and heartburn-free days was reported for PPIs compared to H2RAs (Teragawa et al. 2020). In this clinical trial, the effect of a PPI drug (omeprazole) was compared with three doses of Asvard capsule, an herbal medicine with a combination of *M. communis* and *R. damascena*.

Our finding revealed that omeprazole relieved symptoms and severity of chest pain in participants of the control group. It has been found in several studies that taking omeprazole can treat NCCP and other symptoms associated with GERD. However, some studies indicated that PPIs have an insignificant effect on NCCP. For example, in a systematic review study conducted by Burgstaller et al. (2014), it was found that PPIs were not more effective compared to placebo in the treatment of NCCP (Burgstaller et al. 2014). In addition, Maradey-Romero and Fass reported that higher doses of PPIs and H2RAs could be effective for the treatment of GERD-related NCCP. However, those doses are not safe and can lead to side effects (Maradey-Romero and Fass 2014). The side effects of long-term use of PPIs include bacterial infections, and kidney and respiratory problems, as well as a negative effect on hip fractures (Lahner et al. 2013; Miwa et al. 2015; Vanheel and Tack 2014). Due to the problems of routine treatment with PPIs, 50% of people seek other treatment methods such as complementary therapies and herbal medicine (Sadeghi et al. 2020). Due to the frequent side effects of PPIs, a number of GERD patients believe that the use of these drugs is not safe (Rameau et al. 2021). However, in the present study, few side effects were observed in the omeprazole group. Constipation was the main reported side effect in all the groups of the study, which was in line with the findings of a study by Paknejad et al. on children, where constipation was reported as an important side effect of taking omeprazole as the routine treatment of GERD (Paknejad et al. 2021). However, side effects such as constipation, diarrhea, and headache are considered mild disorders related to PPIs.

In addition, the present study showed a remarkable effect of all three doses of Asvard capsule on reducing the recurrence and severity of NCCP compared to before the intervention. It was also found that the herbal medicine (Asvard) had better results than routine treatment (omeprazole). In line with our findings, Adel Mehraban et al. (2021) presented the remarkable effect of rose oil on reflux and its symptoms such as chest pain, comparing the effect of the routine treatment by omeprazole (Adel Mehraban et al. 2021). Rose is an effective agent in reducing abdominal spasms (Sadraei et al. 2013). Also, this plant has useful compounds such as antioxidants, antimicrobial, antidiabetic, and most importantly, antidepressants and stress relaxers (Emerald 2024). Stress is one of the most important psychological factors in people suffering from GERD and subsequently NCCP. Also, *R. damascena* reduces ileum movements through the stimulation of beta-adrenergic and opioid receptors and calcium channels, which ultimately leads to the relief of digestive disorders (Mahboubi 2016).

Another compound used in the Asvard capsule was *M. communis*, which is a widely used herb in the treatment of digestive disorders. One of the main factors for NCCP is unregulated stomach acid. *M. communis* has a stomach acid regulation effect. Another effect of *M. communis* is on *Helicobacter pylori* eradication, which by controlling the infection can significantly control the symptoms of NCCP related to GERD. Other properties of herbal medicines like *M. communis* that could relieve unknown pain such as NCCP are anti-inflammatory, antifungal, pain relieving, carminative, antiviral, antibacterial, anti-diarrheal, and antioxidant. Additionally, there are flavonoids, procyanidins, anthocyanidins, and phenolic acids in the extract of *M.* *communis*, leading to the improvement of digestion and thus treatment of NCCP-related to GERD (Alipour et al. 2014; Benchikh 2018; Mansour et al. 2022; Touaibia 2017). In several studies, including the study of Jabri et al. (2016) (Jabri et al. 2016), Paknejad et al. (2021) (Paknejad et al. 2021), and Zahlinejad et al. (2016) (Zohalinezhad et al. 2016) the effect of different formulations of *M. communis* in improving reflux and related complications in animal and human samples was recorded. Also, in the sources of traditional medicine, the fruit of the plant in fresh and dried form is known as a useful agent for the treatment of digestive diseases.  *M. communis* can be prescribed as an herbal medicine or as a complementary treatment for a wide range of digestive problems (Akbar and Akbar 2020).

One of the key results of this study was that the effect of the Asvard capsules on the patients of intervention groups was visible after the follow-up period, as two weeks after stopping the intervention, the average score of chest pain remained stable or increased slightly. However, the NCCP average score in the control group increased significantly after two weeks of stopping the treatment, which indicates the recurrence of the disorder. These findings support the results of a study by Salehi et al., in which the recurrence rate was lower in patients who used the *M. communis* L. syrup compared to the omeprazole group (Salehi et al. 2017). In addition, it seems that pharmacological interventions based on traditional and herbal medicines can have a favorable effect with fewer side effects and less possibility of recurrence in patients with GERD and NCCP (Xiao et al. 2018). 

The authors of this article acknowledge the limitation related to the number of participants in the groups, which was associated with the unwillingness of participants. Also, investigating the effects of herbal and chemical medications requires research works with a long period, and the current study with 8 weeks of study was relatively acceptable, however, a longer period is recommended in future studies. The data of this study were not sufficient to identify an optimal dose because all the treatment methods (doses) were effective without disorder recurrence. However, it seems that further biochemical studies and clinical trials with a longer treatment and follow-up period are required to find the most effective dose.

In this study, we compared the effect of Asvard capsules containing the extract of *M. communis* and *R. damascena* in three doses with a PPI medication (Omeprazole) on NCCP. Our findings presented that all the treatment methods were effective in decreasing the severity of chest pain associated with GERD. Besides, the proposed interventions in this study (all the dosages of Asvard capsule) had better results than those of the control one. Also, despite the therapeutic effect in all groups during the study period, an increase in the average NCCP score was observed in the omeprazole group, which is a sign of the recurrence of NCCP symptoms. Therefore, due to the lower score of NCCP symptoms and the non-recurrence of the complication in the intervention groups, it seems that the Asvard capsule could be suggested as a preferable treatment or complementary medicine for NCCP. 
